# Concentrated ambient PM_2.5_ exposure affects mice sperm quality and testosterone biosynthesis

**DOI:** 10.7717/peerj.8109

**Published:** 2019-11-28

**Authors:** Yingying Yang, Tingting Yang, Shengxin Liu, Zhijuan Cao, Yan Zhao, Xiujuan Su, Zehuan Liao, Xiaoming Teng, Jing Hua

**Affiliations:** 1 Department of Women and Children’s Health Care, Shanghai First Maternity and Infant Hospital, Tongji University School of Medicine, Shanghai, China; 2 Department of Medical Epidemiology and Biostatistics, Karolinska Institutet, Stockholm, Sweden; 3 Department of Social Medicine, School of Public Health, Fudan University, Shanghai, China; 4 School of Biological Sciences, Nanyang Technological University, Singapore, Singapore; 5 Department of Microbiology, Tumor and Cell Biology (MTC), Karolinska Institutet, Stockholm, Sweden; 6 Department of Assisted Reproductive Medicine, Shanghai First Maternity and Infant Hospital, Tongji University School of Medicine, Shanghai, China

**Keywords:** Fine particulate matter (PM_2.5_), Sperm quality, Testosterone biosynthesis, Reproductive health

## Abstract

**Background:**

Studies suggested that PM_2.5_ exposure could lead to adverse reproductive effects on male animals. However, the underlying mechanism is still not clear. Besides, animals in the majority of previous studies were exposed to PM_2.5_ through intratracheal instillation which should be improved. In addition, limited amount of research has been conducted in China where the PM_2.5_ concentration is higher and the PM_2.5_ components are different. The aim of this work is to explore the effects of concentrated ambient PM_2.5_ (CAP) on mice sperm quality and testosterone biosynthesis.

**Methods:**

A total of 12 male C57BL/6 mice were exposed to filtered air (FA) or CAP for 125 days using the Shanghai Meteorological and Environmental Animal Exposure System. The mice sperm concentration, sperm motility, DNA fragmentation index, high DNA stainability and plasma testosterone were analyzed. Testicular histology and sperm morphology were observed through optical microscope. Testosterone biosynthesis related gene expressions were analyzed using real-time PCR, including cytochrome P450 CHOL side-chain cleavage enzyme (P450scc), steroidogenic acute regulatory protein (StAR), 3β-hydroxysteroid dehydrogenase (3β HSD), 17β-hydroxysteroid dehydrogenase, cytochrome P450 aromatase (P450arom), estrogen receptor (ER), androgen receptor (AR) and follicle stimulating hormone receptor (FSHR).

**Results:**

Exposure to CAP resulted in disturbance of various stages of spermatogenesis and significant higher percentage of abnormal sperm (FA vs. CAP: 24.37% vs. 44.83%) in mice testis. CAP exposure significantly decreased sperm concentration (43.00 × 10^6^ vs. 25.33 × 10^6^) and motility (PR: 63.58% vs. 55.15%; PR + NP: 84.00% vs. 77.08%) in epididymis. Plasma testosterone concentration were significantly declined (0.28 ng/ml vs. 0.69 ng/ml) under CAP exposure. Notably, the levels of testosterone biosynthesis related genes, StAR, P450scc, P450arom, ER and FSHR were significantly decreased with CAP exposure.

**Conclusion:**

Concentrated ambient PM_2.5_ exposure altered mice sperm concentration, motility and morphology, which might be mediated primarily by the decline in testosterone concentration and testosterone biosynthesis process.

## Introduction

Unprecedented growth and development in China have had a substantial cost on the environment and pose a threat to public health. Air pollution is a major issue in China, and smog is increasing severely in many cities (Liu, Xu & Yang, 2018). Ambient fine particulate matter (PM_2.5_, aerodynamic diameter ≤2.5 μm) is one of the most important air pollutants, which can carry different compounds, including organic elements, biological species, metals and environmental chemicals such as polycyclic aromatic hydrocarbons (Javed et al., 2019; Peng et al., 2019). PM_2.5_ can enter the gas exchange region in the lungs (Peng et al., 2016), causing various lung diseases and cancer (Gonzalez-Molina et al., 2019; Li, Zhou & Zhang, 2018; Liao, Chua & Tan, 2019; Liao et al., 2019; Phua et al., 2018; Tomczak et al., 2016). Studies have shown that PM_2.5_ exposure adversely affects the hypothalamic pituitary axis and testicular spermatogenesis which could potentially cause sperm alterations (Jeng & Yu, 2008).

Human sperm quality has declined worldwide in the last few decades and research on the causes of this continuing decline is urgently needed (Levine et al., 2017). Environmental pollutants affect the male reproductive system of human and animals negatively (Sifakis et al., 2017). Both human observational epidemiology studies and animal experiments support the hypothesis that air pollutants cause defects during gametogenesis, leading to a drop in reproductive capacity in exposed populations.

Human epidemiological studies have shown that ambient PM_2.5_ levels are negatively associated with testosterone levels, sperm count and motility, and positively associated with abnormalities in sperm morphology (Hammoud et al., 2010; Jurewicz et al., 2015; Lao et al., 2018; Zhou et al., 2014). Animal studies also showed PM_2.5_ exposure could lead to adverse reproductive effects on male animals, but a majority of the animals in previous studies were exposed to PM_2.5_ through intratracheal instillation which is different from natural exposure and thus, may not illustrate the impact of PM_2.5_ exposure on health. Moreover, the average PM_2.5_ concentration was (71.6 ± 33.2) μg/m^3^, which was representative for the PM_2.5_ levels in USA (Qiu et al., 2018). However, the average annual concentrations of PM_2.5_ in USA was much lower than that in China, for instance, 7.94 μg/m^3^ in USA and 41.62 μg/m^3^ in China in 2017 (Yang et al., 2018). In addition, the chemical components of PM_2.5_ in different regions vary dramatically, which could cause different health effects (Snider et al., 2016). Further studies are needed in China where with higher concentration and different chemical components of PM_2.5_.

In the present study, the impact of concentrated ambient PM_2.5_ (CAP) exposure on C57BL/6 mice sperm quality and testosterone biosynthesis was explored using the Shanghai Meteorological and Environmental Animal Exposure System (“Shanghai-METAS”).

## Materials and Methods

### Animal exposure to PM_2.5_

A total of twelve 6-week-old male C57BL/6 mice were obtained from Shanghai Lingchang Biotech Limited Company (Certification No. 2013001821608). After 1-week adaption, the mice were randomly divided into two groups of size 6 (exposed to filtered air (FA); CAP, exposure to concentrated ambient PM_2.5_), respectively.

Mice were exposed to FA or CAP using Shanghai Meteorological and Environmental Animal Exposure System, “Shanghai-METAS” (patent #201510453600.8-), which has been described previously (Du et al., 2018). The concentrated PM_2.5_ was generated using the modified versatile aerosol concentration enrichment system (VACES) (Geller et al., 2005; Maciejczyk et al., 2005), which uses the principle of the condensational growth of the ambient particles followed by virtual impaction to concentrate the aerosol (Sioutas, Kim & Chang, 1999). For the filter air chamber, a high-efficiency particulate-air filter (Shanghai Lianbing Environmental Protection Technology Co. Ltd., PN#H3) was used to remove the ambient particulate matter from the ambient air (Laing et al., 2010). TEOM1405 (Thermo, Waltham, MA, USA) was used to measure the real-time concentrations of PM_2.5_, and sample of PM_2.5_ was simultaneously collected on filters to determine the accurate concentrations. The “Shanghai-METAS” is located in the school of Public Health at Fudan University at Xujiahui District in Shanghai where most of the ambient PM_2.5_ is attributed to traffic exhaust. The light cycle was 12 h light/12 h dark, the temperature was 18–25 °C, and the relative humidity was 40–60% in the living environment of “Shanghai-METAS.” The duration of exposure was 8 h per day, 7 days per week for 125 days. Mouse euthanasia and tissue collection were performed on the day following the last exposure.

The Animal Experimental Ethics Committee of the Department of Laboratory Animal Science, Fudan University (ethics reference number: 201805003Z) approved this study. All animals were treated humanely and with regards to alleviation of suffering.

### Testicular pathological analysis

The fresh isolated testicle tissues of mice were fixed in Bouin’s solution. The fixed tissues were then dehydrated and processed for paraffin embedding. The paraffin was sectioned about five μm thick and further stained using hematoxylin-eosin. Morphological changes were observed under a microscope (Luo et al., 2018). A pathologist, blinded to the sample groupings, was hired to take images which cover the entire testicular tissues of each testis in two consecutive sections.

### Sperm morphology analysis

The sperm morphology was assessed using SpermBlue staining method (Microptic SL, Barcelona, Spain) according to Van Der Horst & Maree (2009). Pipetted 10 μl of sperm sample on the edge of the slide and drag the drop with a second slide following 45° angle. Waited for 20–30 s at room temperature. Dropped the slide into deionized water twice. Located the slide on a vertical position and left it to air dry at room temperature. Stained slides were used to perform morphology evaluation using the morphometry module of the Sperm Class Analyzer (Microptic SL, Barcelona, Spain). A total of 200 sperms were analyzed (Ilgin et al., 2017).

### Sperm concentration and motility analysis of epididymis

The organ coefficients of testis were calculated using the weights of freshly isolated testes and normalized to the animal’s weight. Using micro-scissors, six deep cuts were made in each cauda of the left epididymis, releasing sperm into a medium of one ml normal saline. After incubation at 37 °C for 15 min, nylon mesh (pore size: 70 μm) was used to filter the suspension. A total of 10 μl semen was obtained to assess sperm concentration, progressive motility and total sperm motility using a computer assisted sperm analysis system (Krause, 1995).

### Sperm chromatin structure assay

Sperm DNA fragmentation index (DFI) and high DNA stainability (HDS) were assessed by the sperm chromatin structure assay, which is the most widely used test for sperm DNA damage (Panner Selvam & Agarwal, 2018). Basic protocol steps were described previously (Evenson, 2013). Fresh semen was thawed in a 37 °C water bath and diluted to 1–2 × 10^6^ sperm/ml with TNE buffer. A total of 200 ul of this sperm suspension were added to a test tube to which 400 ul of acid detergent solution was added. After 30 s, 1.2 ml of acridine orange dye staining solution was added to the sample, and then flow cytometric measurement commences.

### Detection of the level of testosterone in plasma

The level of testosterone in plasma was tested using enzyme-linked immunosorbent assay kit following the manufacturer’s instructions. Briefly, all reagents, samples, controls and standards were prepared as instructed. Samples, standards and controls were added into wells and prepared labeled HRP-Conjugate was added to each well. After incubation at 37 °C and washing, 3,3′,5,5′-Tetramethylbenzidine substrate solution was added to each well. Incubated the plate at room temperature and added stop solution (Sulphuric acid, 0.15 mol/l) to each well. Read the mean absorbance of the solution per well using a microplate reader at 450 nm within 15 min of stopping the reaction. The concentration of testosterone per well was generated by comparing values per sample with the appropriate standard curve (Miller et al., 2013).

### Real-time PCR

Primer Premier 5.0 software was used to design primers for this study (Table S1), based on GenBank sequence of target genes including cytochrome P450 CHOL side-chain cleavage enzyme (P450scc), steroidogenic acute regulatory protein (StAR), 3β-hydroxysteroid dehydrogenase, 17β-hydroxysteroid dehydrogenase, cytochrome P450 aromatase (P450arom), estrogen receptor (ER), androgen receptor (AR), follicle stimulating hormone receptor (FSHR) and glyceraldehyde 3-phosphate dehydrogenase. Total RNA was isolated from testis with TRIzol^®^ reagent (Invitrogen, Waltham, MA, USA), and then reverse Strand cDNA PrimeScripttm RT reagent Kit with gDNA Eraser (TaKaRa Bio, SKU: RR047A) according to the manufacturer’s instructions. Quantitative PCR was performed using Promega GoTaq^®^ qPCR Master Mix (Promega Corporation, Madison, WI, USA, CAT#: A6001) and ABI VIIA 7 Real Time PCR system (Applied Biosystem, Carlsbad, CA, USA). The specificity of the PCR products was performed using melting curve analyses. Relative gene expression levels were calculated as 2^−ΔΔCt^.

### Statistical analyses

IBM SPSS statistics 23.0 software was used for statistical analysis. The results were expressed as means ± standard deviation (SD). Student’s *t*-test was used to compare the differences between two groups for variables with normal distribution, otherwise the Wilcoxon signed-rank test was used. *P*-value < 0.05 was considered as statistically significant.

## Results

### PM_2.5_ exposure concentration and testis weights

During the 125 days intervention time, the ambient PM_2.5_ concentration in Shanghai during the exposure time was (36.57 ± 17.46) μg/m^3^. The average concentrations of PM_2.5_ were (9.86 ± 13.83) μg/m^3^ in the FA cage and (153.05 ± 33.58) μg/m^3^ in the CAP cage, respectively. We observed no statistical differences in body weight, testis weight or relative weight between the two groups of mice (Table 1).

**Table 1:** Organ weights of C57BL/6 mice exposed to PM_2.5_.

Group	Sample size	Body weight (g)	Testis weight (g)	Relative weight (Testis/body weight) (g/100 g)
FA	6	28.05 ± 1.20	0.24 ± 0.02	0.83 ± 0.07
CAP	6	28.27 ± 1.57	0.24 ± 0.01	0.87 ± 0.06
*P*-value	–	0.788	0.379	0.407

**Note:**

FA, filtered air; CAP, concentrated ambient PM_2.5_. Students’ *t*-test were used to test the difference between the two groups.

### CAP exposure and testicular histology

**Figure 1:**
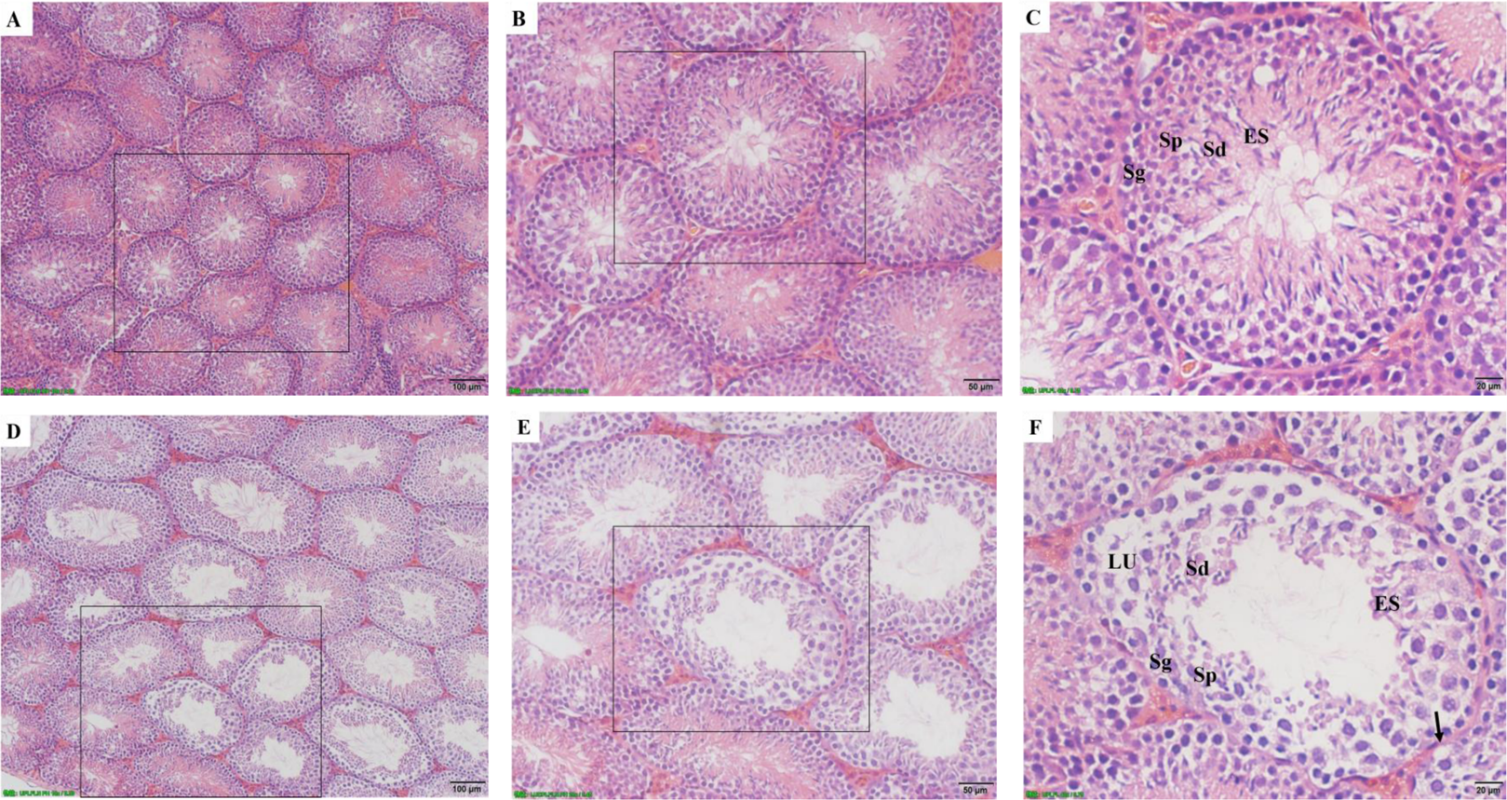
The effects of CAP exposure on seminiferous tubule morphology in testis. (A) Seminiferous tubule morphology in testis of FA group; (B) the magnification image of the selected area from A; (C) the magnification image of the selected area from B. A, B and C showing normal spermatogenesis with normal features of spermatogonia (Sg), Spermatocytes (Sp), spermatids (Sd), elongated spermatids (ES); (D) seminiferous tubule morphology in testis of CAP group; (E) the magnification image of the selected area from D; (F) the magnification image of the selected area from E. D, E and F exhibiting damage on tubules and spermatogenesis. The basal lamina degenerated with less basal cells. Spermatogenesis stopped at the primary spermatocyte stages as seen in the lumen (LU). The germ cells showed overall decrease in cytoplasmic ground substance followed by vacuolization (arrow). A and D, 100×; B and E, 200×; C and F, 400×.

To investigate potential mechanisms whereby exposure to CAP reduces sperm count in the epididymis (Table 2), pathological assessments were performed on the testis of FA- and CAP-exposed mice. Figure 1 showed the difference of seminiferous tubules in testis of FA group (Figs. 1A–1C) and CAP group (Figs. 1D–1F). In the FA group, the seminiferous tubules in testis showed the different stages of spermatogenesis including spermatogonia (Sg), Spermatocytes (Sp), spermatids (Sd) and elongated spermatids in normal appearance. The mice exposed to CAP showed disturbance in the various stages of spermatogenesis. The basement membrane and tunic propria became thin and disrupted. Germ cells reduced in overall numbers and vacuolization were observed.

**Table 2:** Sperm parameters in different mice group.

Group	Sample size	Sperm concentration (×10^6^)	PR (%)	PR + NP (%)	DFI (%)	HDS (%)
FA	6	43.00 ± 10.80	63.58 ± 4.83	84.00 ± 2.43	2.96 ± 1.14	0.90 ± 0.18
CAP	6	25.33 ± 9.39	55.15 ± 3.83	77.08 ± 3.22	4.39 ± 1.56	1.52 ± 0.66
*P*-value	–	0.013	0.007	0.002	0.100	0.074

**Note:**

FA, filtered air; CAP, concentrated ambient PM_2.5_; PR, progressive motility; NP, non-progressive motility; DFI, DNA fragmentation index; HDS, high DNA stainability. Students’ *t*-test were used to test the difference between the two groups.

### CAP exposure and sperm morphology

**Figure 2:**
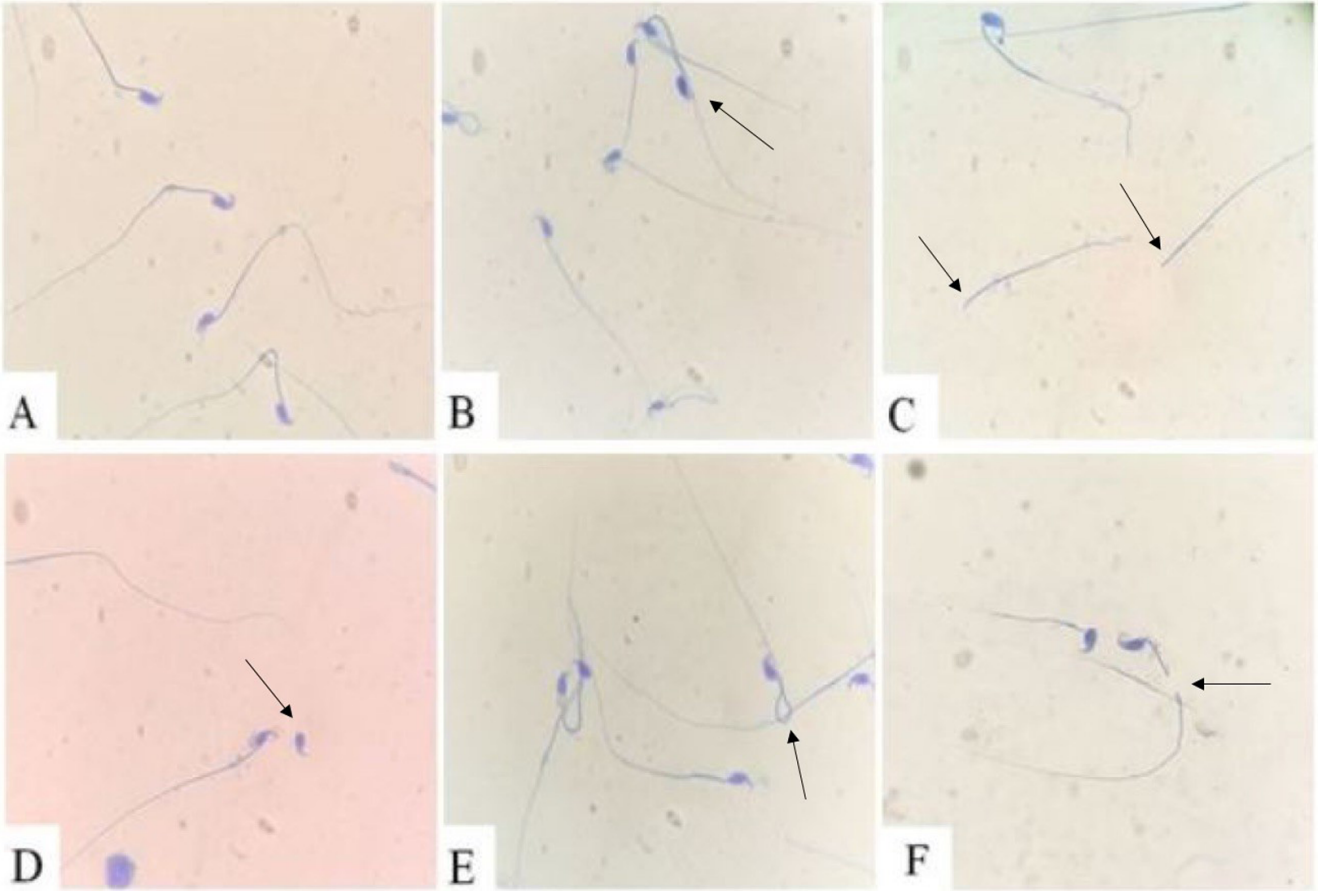
Sperm morphology. (A) Normal sperm; (B) two tails defect; (C) no-head defect; (D) no-tail defect; (E) coiled tail defect; (F) bent tail defect.

As shown in Fig. 2, all types of defective sperms were found in both groups, including multiple tails, no-head, no-tail, coiled tail, bent tail. However, the percent of abnormal sperm in the CAP group was (44.83 ± 5.18)%, which was significantly higher than in FA group (24.37 ± 5.96)%, *P* < 0.001.

### Sperm parameters

As shown in Table 2, the sperm concentration in epididymis of the CAP group was significantly lower than that of the FA group. The progressive motility percent and total motility (progressive motility (PR) and non-progressive motility (NP) percent) of the CAP group were significantly lower than that of the FA group. The DFI and HDS of the CAP group were higher than that of FA group, but the differences were not significant.

### Testosterone level in plasma and testosterone biosynthesis related mRNA expression

**Figure 3:**
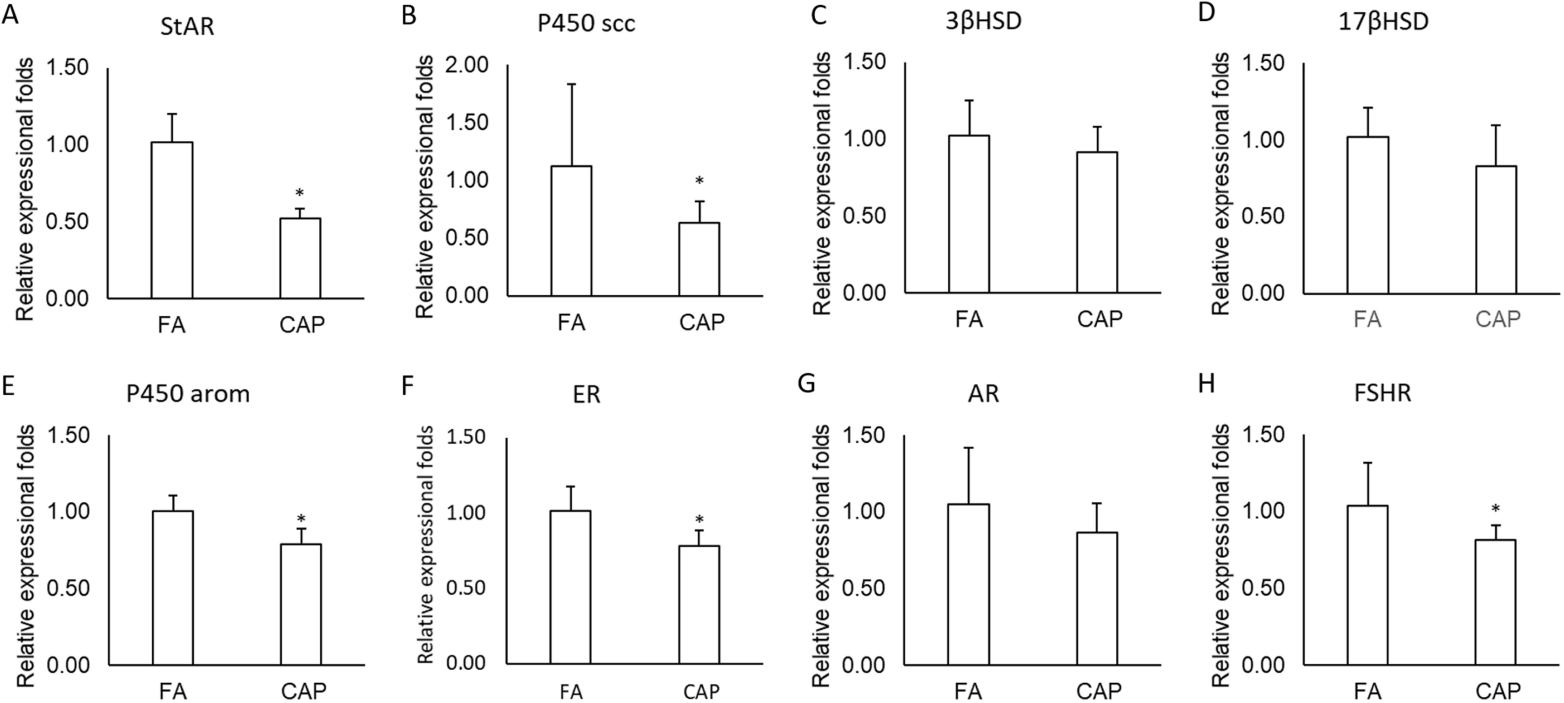
The expressions of testosterone synthesis and function related genes. (A) StAR. (B) P450 scc. (C) 3βHSD. (D) 17βHSD. (E) P450 arom. (F) ER. (G) AR. (H) FSHR. The data are expressed as the (mean ± SD). *N* = 6 per group. *P < 0.05 compared with FA group. Abbreviations: FA, filtered air; CAP, concentrated ambient PM_2.5_. Students’ *t*-test were used to test the difference between the two groups.

Testosterone is central in the regulation of spermatogenesis. To test if CAP exposure impacts spermatogenesis through alteration of reproductive hormone production, we assessed plasma testosterone levels. The average concentrations of testosterone in plasma in the CAP group were (0.28 ± 0.10) ng/ml, which was significantly lower than in FA group (0.69 ± 0.10) ng/ml (*P* < 0.001). As shown in Fig. 3, the mRNA expressions of StAR, P450scc, P450arom, ER and FSHR in CAP groups were significantly lower than in FA group (Figs. 3A, 3B, 3E, 3F and 3H), but not 3βHSD, 17βHSD and AR (Figs. 3C, 3D, and 3G).

## Discussion

The level of ambient PM_2.5_ in many Chinese areas is higher than the recommended standards of World Health Organization (WHO). Studies have shown that inhaled PM_2.5_ can lead to cardiovascular, respiratory and other system damage (Bowe et al., 2018; Fu et al., 2019; Jiang et al., 2018; Khan et al., 2019; Liu et al., 2017; Phua et al., 2018; Pun et al., 2017). A limited number of animal toxicological studies have provided evidence of association between exposure to PM_2.5_ and the deterioration of semen quality. The biological mechanisms linking ambient air pollution to decreased sperm quality have yet to be determined (Jurewicz et al., 2018).

The present study explored the effects of CAP exposure on male mice using the “Shanghai-METAS.” Our main results are that exposure to CAP (1) disturbs the various stages of spermatogenesis; (2) alters sperm morphology and increases the percentage of abnormal sperm; (3) reduces sperm concentration and motility in the epididymis; (4) decreases the testosterone level in plasma; (5) decreases the expression of testosterone biosynthesis related genes, including StAR, P450scc, P450arom, ER and FSHR. These data collectively suggest that long-term exposure to ambient PM_2.5_ impairs male mice reproduction structure and function.

Testis is one of the most important male reproductive glands, which produce gametes and secrete hormones, primarily testosterone. The present study found that, exposure to high concentrations of PM_2.5_ disturbed various stages of spermatogenesis, damages the basement membrane and tunic propria, as well as reduced number of germ cells. Semen analysis evaluates certain characteristics of a male’s semen and the sperm contained therein. It is done to help evaluate male fertility, whether for those seeking assisted reproduction or verifying the success of vasectomy. In the current study, Sperm parameters, including sperm concentration and motility, declined under concentrated PM_2.5_ exposure. Spermatogenesis is primarily regulated by testosterone (Chao & Page, 2016; O’Hara & Smith, 2015). In the healthy male body, the hypothalamic-pituitary-gonads axis is regulated by testosterone (Anderson, Kinniburgh & Baird, 2002). Our study found that the circulating testosterone decreased after exposure to CAP. Our results were consistent with previous animal studies showing that intratracheal instillation of ambient PM_2.5_ or inhalation of diesel exhaust significantly increased the proportion of abnormal sperm cells, decreased sperm count and testosterone level (Cao et al., 2015; Watanabe & Oonuki, 1999).

Steroidogenic acute regulatory protein was identified as a principal protein with cholesterol transport across the mitochondrial membrane (Papadopoulos et al., 2006). The leydig cell steroidogenic pathway in the rat is ordered P450scc-3βHSD-CYP17-17βHSD, which synthesize, respectively, pregnenolone-progesterone-17α-hydroxyprogesterone/androst-enedione-testosteone (Haider, 2004). Pituitary-derived FSH provides indirect structural and metabolic support for development of spermatogonia into mature spermatids via its membrane-bound receptor (FSHR) in Sertoli cells (Oduwole, Peltoketo & Huhtaniemi, 2018). Androgens are necessary for normal male phenotype expression, including the outward development of secondary sex characteristics and the initiation and maintenance of spermatogenesis (McLachlan et al., 2002). AR itself also plays an important role in the feedback regulation of testosterone levels (Solakidi et al., 2005). Mammalian sperm capacitation, acrosome reaction and fertilizing ability are stimulated by estradiol and environmental estrogens (Beato, Herrlich & Schütz, 1995), which mediates by estrogen receptors (Gustafsson, 1999). The present study found that exposure to CAP decreased testosterone biosynthesis related genes, including StAR, P450scc, P450arom, ER and FSHR. The present findings are consistent with previous study results (Qiu et al., 2018). These findings suggest that exposure to CAP by inhalation lead to impaired spermatogenesis, and the impairment might be mediated primarily by the reduction in testosterone biosynthesis.

Notably, different from the previous studies by intratracheal instillation, the mice in the current study were exposed to CAP or filter air using a whole body exposure system, which mimic real-world exposure to environmentally relevant PM_2.5_ or FA (Wang et al., 2017; Ying et al., 2014). In addition, the VACES has been tested and validated in previous study (Maciejczyk et al., 2005), which was one of the advantages of present study. Interestingly, the concentration of CAP in our study (153.05 μg/m^3^) was more than two times higher than the one conducted in USA (71.6 μg/m^3^) (Qiu et al., 2018). With CAP exposure, the percentage of abnormal sperm from the current study (44%) was higher than that in previous study (34%). Besides, the concentration of plama testosterone was lower than that in previous study (0.28 ng/ml vs. 1.2 ng/ml). However, when comparing these two studies, typical dose-response effect was not observed between CAP and abnormal sperm rate as well as circulating testosterone. Further animal studies exploring the dose-response effects of CAP on reproductive function are warranted.

## Conclusions

This study, using a whole body PM_2.5_ exposure system, demonstrates that CAP exposure alter mice sperm concentration, motility and morphology, which might be mediated primarily by the decline in testosterone concentration and testosterone biosynthesis processes.

## Supplemental Information

10.7717/peerj.8109/supp-1 Supplemental Information 1 The primers of the analyzed genes.

10.7717/peerj.8109/supp-2 Supplemental Information 2 Raw data of sperm parameters.

10.7717/peerj.8109/supp-3 Supplemental Information 3 Raw data of mRNA expression.

10.7717/peerj.8109/supp-4 Supplemental Information 4 Detail procedures of Real Time-PCR.

## Additional Information and Declarations

### Data Availability

The following information was supplied regarding data availability: The raw data are available in Supplemental Files.
