# Supplementary material for: Concentrated ambient PM2.5 exposure affects mice sperm quality and testosterone biosynthesis
Source: PeerJ. 2019 Nov 28;7:e8109. doi: 10.7717/peerj.8109 (PMC6885350; doi:10.7717/peerj.8109)
Supplement: Supplemental Information 1 [file peerj-07-8109-s001.docx]

**Supplementary Table 1. The primers of the Analyzed Genes**

| **Gene** | **Forward primer** | **Reverse primer** |
| --- | --- | --- |
| GAPDH | 5’-TGAACGGGAAGCTCACTGG-3’ | 5’-TCCACCACCCTGTTGCTGTA-3’ |
| P450scc | 5’-AGGTCCTTCAATGAGATCCCTT-3’ | 5’-TCCCTGTAAATGGGGCCATAC-3’ |
| StAR | 5’-ATGTTCCTCGCTACGTTCAAG-3’ | 5’-CCCAGTGCTCTCCAGTTGAG-3’ |
| 3β HSD | 5’-CCTCCGCCTTGATACCAGC-3’ | 5’-TTGTTTCCAATCTCCCTGTGC-3’ |
| 17β HSD | 5’-ACTTGGCTGTTCGCCTAGC-3’ | 5’-GAGGGCATCCTTGAGTCCTG-3’ |
| P450arom | 5’-ATGTTCTTGGAAATGCTGAACCC-3’ | 5’-AGGACCTGGTATTGAAGACGAG-3’ |
| ER | 5’-GCCACATAGTCAACCTTGCAGC-3’ | 5’-CGTCTTCTGCTCCACATAGAGC-3’ |
| AR | 5’-CTGGGAAGGGTCTACCCAC-3’ | 5’-GGTGCTATGTTAGCGGCCTC-3’ |
