# Supplementary material for: Concentrated ambient PM2.5 exposure affects mice sperm quality and testosterone biosynthesis
Source: PeerJ. 2019 Nov 28;7:e8109. doi: 10.7717/peerj.8109 (PMC6885350; doi:10.7717/peerj.8109)
Supplement: Supplemental Information 4 [file peerj-07-8109-s004.docx]

**Details of Real-time PCR**

Primer Premier 5.0 software was used to design primers for this study (supplementary Table 1), based on GenBank sequence of target genes including cytochrome P450 CHOL side-chain cleavage enzyme (P450scc), steroidogenic acute regulatory protein (StAR), 3β-hydroxysteroid dehydrogenase (3β HSD), 17β-hydroxysteroid dehydrogenase (17β HSD), cytochrome P450 aromatase (P450arom), Estrogen receptor (ER), androgen receptor (AR), follicle stimulating hormone receptor (FSHR) and glyceraldehyde 3-phosphate dehydrogenase (GAPDH).

**Supplementary Table1. The primers of the Analyzed Genes**

| **Gene** | **Forward primer** | **Reverse primer** |
| --- | --- | --- |
| GAPDH | 5’-TGAACGGGAAGCTCACTGG-3’ | 5’-TCCACCACCCTGTTGCTGTA-3’ |
| P450scc | 5’-AGGTCCTTCAATGAGATCCCTT-3’ | 5’-TCCCTGTAAATGGGGCCATAC-3’ |
| StAR | 5’-ATGTTCCTCGCTACGTTCAAG-3’ | 5’-CCCAGTGCTCTCCAGTTGAG-3’ |
| 3β HSD | 5’-CCTCCGCCTTGATACCAGC-3’ | 5’-TTGTTTCCAATCTCCCTGTGC-3’ |
| 17β HSD | 5’-ACTTGGCTGTTCGCCTAGC-3’ | 5’-GAGGGCATCCTTGAGTCCTG-3’ |
| P450arom | 5’-ATGTTCTTGGAAATGCTGAACCC-3’ | 5’-AGGACCTGGTATTGAAGACGAG-3’ |
| ER | 5’-GCCACATAGTCAACCTTGCAGC-3’ | 5’-CGTCTTCTGCTCCACATAGAGC-3’ |
| AR | 5’-CTGGGAAGGGTCTACCCAC-3’ | 5’-GGTGCTATGTTAGCGGCCTC-3’ |

Total RNA was isolated from testis with TRIzol® reagent (Invitrogen, USA), and then reverse Strand cDNA PrimeScript^tm^  RT reagent Kit with gDNA Eraser (TaKaRa Bio, SKU: RR047A) according to the manufacturer’s instructions. Quantitative PCR was performed using Promega GoTaq® qPCR Master Mix (Promega Corporation, Madison, WI, USA, CAT#: A6001) and ABI VIIA 7 Real Time PCR system (Applied Biosystem, Carlsbad, CA, USA).

The reverse transcription product was diluted by 5 times.

The PCR reaction system:

| GoTaq®qPCR Master Mix, 2X | 5 ul |
| --- | --- |
| ddH_2_O | 3 ul |
| Primer F | 0.75 ul |
| Primer R | 0.75 ul |
| DNA Sample | 0.5 ul |
| Total Volume | 10 ul |

The reaction conditions were as below.

| 95℃ | 10min | 1 cycle |
| --- | --- | --- |
| 95℃ | 15s | 40cycles |
| 60℃ | 30s | 40cycles |
| 72℃ | 30s | 40cycles |
| 72℃ | 10min | 1cycle |

The specificity of the PCR products were performed using melting curve analyses. Relative gene expression levels were calculated as 2^-ΔΔCt^.
